# Assessment of Nutrition Status in Amateur Windsurfers during Regattas in the Competitive Period—A Field Study

**DOI:** 10.3390/ijerph18126451

**Published:** 2021-06-15

**Authors:** Anna Gogojewicz, Barbara Pospieszna, Jakub Bartkowiak, Ewa Śliwicka, Joanna Karolkiewicz

**Affiliations:** 1 Department of Food and Nutrition, Poznan University of Physical Education, 61-871 Poznań, Poland; gogojewicz@awf.poznan.pl (A.G.); qbabartkowiak@gmail.com (J.B.); karolkiewicz@awf.poznan.pl (J.K.); 2 Department of Athletics, Strength and Conditioning, Poznan University of Physical Education, 61-871 Poznań, Poland; bpospieszna@awf.poznan.pl; 3 Department of Physiology and Biochemistry, Poznan University of Physical Education, 61-871 Poznań, Poland

**Keywords:** physical activity, sports nutrition, athletes

## Abstract

Windsurfing is a demanding activity that requires a high level of physical fitness as well as appropriate training and nutritional strategies. Therefore, the aim of this study was to assess the dietary intake of amateur windsurfers and consider possible dietary mistakes. This field study was conducted among 10 Polish male amateur windsurfers (aged 22 ± 2 years, mean training experience of 9.5 ± 4 years). Dietary intake was assessed using a standardized 3-day food record. The total energy expenditure of each participant was estimated using a mobile fitness application. The daily energy supply in assessed portions of the windsurfers’ food was lower than the estimated demand during the competition. The contribution of macronutrients to the total energy intake adhered to those guidelines, but not with the ones recommended for athletes practicing extreme sports. Daily fluid consumption was insufficient. In the windsurfers’ diet, we noticed low consumption of vitamin D and calcium, while cholesterol, sodium, potassium, and phosphorus intake was too high. Nutritional practices of amateur windsurfers during the competitive period do not comply with current sports nutrition guidelines. The results suggest that windsurfers are in need of nutritional education and dietary counseling in order to meet macronutrient intake targets.

## 1. Introduction

Windsurfing is an extremely demanding sport in terms of metabolic requirements as it combines surfing and sailing activity [1]. Windsurfing was, until recent changes in sailing rules allowed sail pumping (PB) during the race, considered as a moderately intense activity [2,3].

Due to those changes, the World Sailing (formerly International Sailing Federation—ISAF) requested its Medical Commission to investigate the physical demands of sail pumping in Olympic-class windsurfing to provide specific guidelines and training recommendations for competitors and coaches. The study by Guével et al. [4] showed that windsurfing is now a much more demanding activity and therefore requires, apart from a high level of physical fitness, very strict sports medicine supervision in terms of both training and nutritional strategies.

A complete training program for windsurfers should include three main elements: (1) highly intense interval training with work and rest periods closely resembling the pumping and relaxation periods; (2) moderately intense continuous training, which improves cardiovascular fitness and local muscle oxidative capacity, and (3) strength training, especially of the upper body muscles, to prepare for the explosive movements, with pumping the most frequent [5].

Sail pumping became one of the most important elements in windsurfing and at the same time the most energetically demanding. It is a repeated action used during the major part of the race, especially in light to moderate wind conditions, in which the athlete rhythmically pulls and pushes the sail to reach a higher speed of the board [5,6]. This non-controllable aspect of boardsailing directly influences the energy cost of windsurfing, which is demonstrated by higher mean heart rate (HR) values measured in male windsurfers during the simulated race in light compared to moderate wind conditions (87 ± 4% vs. 83 ± 5% HRmax) [4]. Apart from wind conditions, additional factors that significantly impact the windsurfers’ energy expenditure are the size of the sail, buoyancy of the board, type of wetsuit, ambient and water temperature, and the level of insolation [6,7].

There are several windsurfing classes, with varying sail sizes, board buoyancies and other individual features (e.g., the use of foil). Slalom is one of the windsurfing classes announced for the Professional Windsurfers Association (PWA) in 2005 to increase the attractiveness of this discipline [8]. In slalom racing, there are multiple very short heats (around 3–5 min long; 2000 m of total course length) performed very close to the beach, always with limited on-water waiting time [9].

In northern countries, yearly training plan of windsurfers is divided, due to weather conditions, into two phases: (1) out-of-water training in the winter and (2) sailing training during warmer months. The competitive phase of the annual training plan consists of a series of regatta connected with the Polish Cup, organized by the Polish Windsurfing Association [10]. The points obtained from each start influence the final result of the competitor. Therefore, for windsurfing slalom class in Poland, competitive season starts at the beginning of May and ends in mid-October.

Because of the long training sessions (athletes spend daily up to 6 h afloat), very restricted consumption possibilities (constant four-limb activity), and practically no storage space to keep food and fluids, nutrition in windsurfing becomes a crucial issue. To meet hydration and energy intake demands, windsurfers administer longer breaks during the training day. Yet, in order to obtain better results, windsurfers spend much time practicing and experimenting with equipment, both on- and off-land, though they do not seem to spend adequate time on their nutrition [11].

We would like to underline that there are no data evaluating nutrition among windsurfers. Therefore, the purpose of this study was the assessment of customary dietary intake in a selected group of amateur athletes participating in national championships in windsurfing slalom.

## 2. Materials and Methods

### 2.1. Study Group

This field study was conducted among 10 Polish amateur male windsurfers, aged 22 ± 2 years, performing windsurfing slalom, with the mean training experience of 9.5 ± 4 years. They were volunteers who responded to the invitation to participate in the study. All study participants were characterized by a good state of physical health, no injuries or chronic conditions, non-smoking, and not taking any medications or dietary supplements. The study was conducted before and during one of the main regattas in the competitive phase of the 2017/18 season—national championships in windsurfing slalom held from 21 to 23 September.

All subjects gave their written consent before taking part in the study. The study protocol was approved by the Local Ethics Committee at the Poznań University of Medical Sciences, reference numbers 150/14 and was self-funded. Data collection complied with the Helsinki declaration for biomedical research on human subjects.

### 2.2. Anthropometric and Body Composition Measurements

Measurements of body mass and height were performed using a medical scale (RADWAG^®^, Radom, Poland). The somatic characteristics of the studied windsurfers are given in Table 1.

### 2.3. Nutritional Assessment

Dietary intake was assessed using a standardized 3-day food record (2 training days and 1 regatta day). The number of meals was expressed in common measurement units (e.g., glass, cup, bowls, spoons, etc.). Sports drinks and other sports foods were included in the questionnaire. The collected information was adjusted based on the atlas of food products prepared by the National Food and Nutritional Institute in Warsaw (Poland). Quantitative analysis of the daily food ration’s composition was performed using the Dietetyk 2016 software package that uses a database prepared by the National Food and Nutritional Institute. Mean intakes of energy and nutrients were compared with current recommendations of the International Society of Sports Nutrition for athletes participating in moderate intensive training (e.g., 2–3 h a day of intense exercise performed 5–6 times a week) or high-volume intensive training (e.g., 3–6 h a day of intense training during 1–2 workouts, 5–6 days a week) who can spend 600–1200 kcal or more per hour during exercise. For this reason, their caloric requirements can reach 40–70 kcal/kg/day (2000–7000 kcal/day for an athlete weighing 50–100 kg) [12].

### 2.4. Daily Energy Expenditure

The total energy expenditure (TEE) of each participant was estimated using the mobile fitness application (Endomondo) with respect to the total training duration, multiplied by the energy expenditure during windsurfing (training: wind 25–35 km/h, air temperature 20–22 °C; regatta: wind 19–25 km/h, air temperature 18–20 °C). Endomondo application allows users to track their fitness activities via GPS from their smartphones. All participants used Samsung’s S-class phones. The results obtained were compared with relevant recommendations proposed by Bernardi et al. [13].

### 2.5. Statistical Analysis

The results are presented as mean values with standard deviations (±SD) and where it is relevant (distribution different from normal and/or high data variability) as medians (Me) and Q1 and Q3 quartiles. To demonstrate a significant difference between the study group and the ISSN and ACSM recommendations, a *t*-test was performed. All descriptive statistics were performed using the STATISTICA 13.0 software package (StatSoft Inc., Tulsa, OK, USA).

## 3. Results

Analyzed amateur windsurfers of slalom class varied in body height, body mass, BMI, as well as in their age and training experience (Table 1).

Obtained dietary results were confronted with dietary recommendations for athletes training sport disciplines of similar character, that is athletes involved in moderate levels of intense training (e.g., 2–3 h per day of intense exercise performed 5–6 times per week) and high volume intense training (e.g., 3–6 h per day of intense training in 1–2 workouts for 5–6 days per week) [14].

The average energy exercise cost of the windsurfers’ ranged from 1250 ± 250 kcal/h in the training day to 1500 ± 250 kcal/h during competitions. Daily energy supply in the assessed food rations of the windsurfers was lower as the estimated demand during the competitive period. The contributions of carbohydrate, protein, and fat to total energy intake were 46.6%, 19.3%, and 33.9%, respectively. The percentage contributions of carbohydrate, protein, and fat to total energy intake were not in accordance with the recommended guidelines for such a group of athletes. Daily consumption of liquids by windsurfers was also insufficient (Table 2).

Windsurfers failed to meet the recommended dietary allowance (RDA) or adequate intake (AI) for vitamin D. However, their intakes exceeded the RDAs for sodium, potassium, and phosphorus (Table 3).

## 4. Discussion

The main finding of this study is that the nutritional practices of amateur windsurfers during the competitive period do not comply with current sports nutrition guidelines. According to the relative VO_2_ values obtained during a windsurfing simulator exercise test, male’s windsurfing can be considered as heavy exercise [6,15]. During 40 min windsurfing simulator tests, the energy expenditure averaged 597.115 kcal [15]. Yet, in typical sailing conditions, the intensity of physical effort depends on (1) windsurfing class, (2) sailing technique, and (3) weather conditions [5,11,16,17,18,19,20]. The slalom competition, in contrast to the freestyle/wave, which includes various figures and tricks, involves fewer body movements performed mainly by the upper body muscles [11]. In opposite to sailing, windsurfing in lower wind speeds increases energy demands. When, in order to increase the board’s speed, more “pumping” is necessary [18], the intensity of physical effort ranges 70–80% of maximum oxygen uptake (VO_2_max), heart requires more than 80% of one’s maximum heart rate, and energy expenditure is at the level of 18–20 kcal/min [5,16]. This effort is especially increased during the down-wind leg when pumping time can reach 69% of the total surfing duration [17]. Thermal load, especially cold-water immersion is the additional factor affecting windsurfers’ energy expenditure [19,20]. During regatta, to all mentioned factors of increased energy expenditure, the ones of emotional origin have to be added.

Olympic-class windsurfing can be considered as a high-intensity endurance type of sport that is comparable to other aerobic sporting activities such as rowing [6]. The energy expenditures of athletes of medium-to-high-intensity sports are on average 600–1200 kcal or more per hour of practice, 40–70 kcal/kg/day, or 2000–7000 kcal/day for a 50–100 kg athlete [14]. Yet, the lower level competitors usually have lower levels of energy expenditure. Daily energy intake of slalom windsurfers was shown to average 2635.6 ± 665.7 kcal [11,21]. In our research, the average daily energy intake was 2885.8 ± 435.9 kcal, while the energy expenditure in training days was 1250 ± 250 kcal/h, and on regatta day 1500 ± 250 kcal/h. So, the daily energy expenditure was not always covered by daily energy supply in the assessed food rations, especially during the competition (Table 2). The failure to cover the energy demands during trainings and competitions was previously reported by Felder et al. [22] and Bernardi et al. [13] in America’s Cup sailors and female surfers. However, those scientists claim that such findings may result from various, sometimes contradictory reasons, e.g., (1) the wrong estimation of energy requirements, (2) undereating during dietary record, or (3) underreporting, in fear of being judged for the quantities they normally consume.

According ISSN [12], a normal diet for healthy adult should provide supply of 45–55% CHO [3–5 g/kg body mass/day], 15–20% PRO [0.8–1.2 g/kg body mass/day], and 25–35% fat [0.5–1.5 g/kg body mass/day]). In our study, the contributions of carbohydrate, protein, and fat to total energy intake were 46.6%, 19.3%, and 33.9%, respectively (Table 2), and adhered to those guidelines, but not with the ones recommended for athletes practicing extreme sports [23,24]. The guidelines for CHO intake in short-lasting (<1 h) extreme sports, e.g., windsurfing are of 5–7 g/kg body weight/day [25]. It is recommended before, during, and after regatta day to increase daily carbohydrate intake to 70% of total calories (approximately 8–10 g/kg for 3500 kcal) or even higher in order to prevent the depletion of glycogen stores during successive days of competition. It is especially important when regatta takes place in light-wind conditions, due to glycogen being the predominant energy fuel during sail pumping [5].

According to the American College of Sports Medicine, in athletes of endurance disciplines, the recommended daily protein intake ranges 1.2–1.7 g/kg/day [26]. The average daily protein intake in the studied windsurfers was 1.8 g/kg body weight (Table 2). Windsurfers require a slightly higher share of protein than those in typically endurance disciplines, especially in light to moderate wind conditions, when sail pumping maneuvers are frequently performed [27,28]. In our study, the average amount of fat in windsurfers’ daily diet was 1.46 g/kg body weight, and it was in the upper reference values for this macroelement in endurance athletes [27]. An increased intake of dietary fat and adequate consumption of essential fatty acids are essential for athletes to maintain the energy balance, replenish intramuscular triacylglycerol stores, improve the blood flow and support the anti-inflammatory properties and post-exercise recovery process. Due to improvements in oxygen metabolism omega-3, fatty acids are known to increase the athletes’ strength and endurance [29]. In analyzed competitors, the share of SFA (9.7%), PUFA (5.9%), and MUFA (13.6%) were at the proper level (Table 2). The daily omega-3 intake was 3.4 ± 1.6 g, which accounted for 1.1% of daily energy intake and was in line with the recommendations, whereas the dietary intake of omega-6 fatty acids averaged 13.24 ± 4.9 g (4.1% of daily energy intake) and was found to be insufficient. In contrast, in the analyzed group, we noted very high cholesterol intake, which averaged 544.7 ± 245.9 mg/day. Such a high result was associated with improperly high intake of products rich in cholesterol, such as fat meat species, offal, cold cuts, and pizza (Table 2). This practice was to some extent explained by the sports setting, where healthy, balanced food is rather difficult to obtain. Instead, there are numerous temporary catering services of questionable nutritional quality placed on or near the beach [30]. Even then, we found that average a windsurfer’s daily diet contained 28.6 ± 7.4 g of fiber, which covered the minimal fiber daily demand (Table 2).

Well-balanced diet for athletes should provide at least the recommended dietary allowance (RDA)/adequate intake (AI) for all micronutrients. Among minerals, the insufficient intake of calcium (991.5 ± 392 mg/day) and vitamin D (4.7 ± 3.0 mg/day) was found in studied windsurfers’ diet. Insufficient calcium intake probably resulted from a very low consumption of milk and milk products, as well as mineral waters. At the same time, a high intake of meat and sausages may be the reason for the excessive source of phosphorus (2068.8 ± 409.1 mg/d), which further limited calcium absorption. The supply of other mineral components’ intake, such as magnesium, iron, and potassium, was within the recommended norms (Table 2).

The average intake of sodium in the windsurfers’ daily diet was estimated to be 2886.2 ± 724.7 mg and exceeded the RDAs for this mineral. However, some sports nutrition guidelines encourage higher sodium ingestion during endurance exercise, and much work was done to quantify sweat sodium losses during exercise. Current guidelines for sodium intake do not recommend specific quantities, nor provide justification for the effectiveness of sodium to improve endurance performance [31]. Sodium over-consumption in the studied athletes was probably due to their dietary preferences, as they declared to frequently consume salty snacks [32].

Potassium intake, significant in muscle and nerve function, and, consequently, in athletic performance, was as well above recommended range but it is questionable whether stated-here intakes (4827 ± 1373) could impact the athletes’ performance.

In analyzed time, windsurfers enhanced their diets with sport drinks and other sport foods. That is probably why they exceeded intake of group B vitamins: B1 (2.2 ± 1.2), B6 (3.3 ± 1.1), and B12 (5.6 ± 2.1), but the daily intake of other ones was kept within values recommended for athletes [14].

In windsurfers’ diet, fluid intake is of particular importance. The need for liquids depends mainly on atmospheric conditions, time and type of effort, and the used sailing technique [6]. Sail pumping can exacerbate the already significant water loss during training and competition, particularly in hot temperature conditions. In our research, the daily fluid intake of 2130.1 ± 400.9 g and was too low (Table 2) [33]. A similar habit has been reported by dinghy sailors and was attributed to limited space for food and fluid storage. Slater and Tan [34], who monitored body mass changes and nutrient intake of sailors during a club regatta, showed that most participants were in the negative fluid balance after racing, most likely due to low voluntary fluid intake. It seems that in windsurfers, the problem of negative fluid balance is even bigger due to the absolute lack of space and the constant four-limb work during sailing.

This study has some limitation. The number of participants is low, but they were only these, who responded to the invitation to participate in the study. It should be noted that limited sample size renders the results lacking adequate external validity. It cannot be excluded that a too low diet’s energy intake may be due to underestimation and/or inaccurate recording of the products consumed by the windsurfers. However, the authors of the study tried to minimize the risk of this limitation by educating the athletes in the aspect of proper diet recording and constant contact with them in this regard. Moreover, extended interpretation of long-term nutritional disparities requires further consideration of various nutrient–nutrient interactions as well as food interactions on bioavailability, supported by complementary data on actual nutritional status at baseline as well as during and after the study. In addition, in our study only amateur male windsurfers participated; hence, potential results extrapolation to elite windsurfers competitors should be treated with caution.

## 5. Conclusions

To conclude, in our study the windsurfers’ diet points to nutritional errors that can lead to a deterioration in nutritional status and performance. A large supply of saturated fatty acids has been observed in the diet of athletes, which was associated with the excessive consumption of animal origin protein products. Moreover, the carbohydrate content indicates that they are consuming less than recommended amounts of this macronutrient. We recommended that their diet should be more varied and enriched with fresh, nutrient-rich whole grains, legumes, fruits, and vegetables. Therefore, nutritional education should be conducted and athletes should be supported in making the right nutritional choices.

Based on the data collected, it is suggested that more research is needed, in larger groups and for both sexes, to obtain more detailed information on the nutrition of windsurfers.

## Figures and Tables

**Table 1:** Demographic and anthropometric data of the windsurfers (*n* = 10).

	Mean ± SD
Age (years)	22 ± 1.9
Body height (cm)	177 ± 6.3
Body mass (kg)	75 ± 7.9
BMI (kg/m^2^)	23.9 ± 1.2
Training experience (years)	9.5 ± 3.9

Values are expressed as means ± SD. BMI, body mass index.

**Table 2:** Energy supply and selected nutrients’ intake in the windsurfers’ daily diet.

Nutrient	Mean ± SD	Me; Q1 ÷ Q3	Recommendations ISSN	% RDA	*p*-Value
Energy intake (kcal)	2885.8 ± 435.9	2849.8; 2497.8 ÷ 3260.6	2000–7000		0.00
kcal/kg_bm_/day/	38.6 ± 3.95	38.3; 35.7 ÷ 41.5	40–70		
Fluids (mL)	2130.1 ± 400.9	2226.7; 1642.4 ÷ 414	2500 *		
CHO (g)	354.7 ± 78.8	341.1; 288.1 ÷ 433.3	250–1200 g/day for 50–150 kg		
% energy	46.6 ± 4.1	47.5; 44.4 ÷ 48.1	55		0.00
g/kg _bm_/day	4.73 ± 0.84	4.6; 3.9 ÷ 5.7	5–8	94.4	
PRO (g)	137.4 ± 26.6	132.9; 123.2 ÷ 144.5	60–300 g/day for 50–150 kg		
% energy	19.3 ± 4.2	19.8; 15.2 ÷ 23.1	20		0.63
g/kg_bm_/day	1.83	0.2; 1.6 ÷ 2.1	1.4–1.8	100	
Fiber (g)	28.6 ± 7.4	27.2; 21.0 ÷ 36,4	25 g	100	0.14
FAT (g)	109.4 ± 21.1	108.5; 89.3 ÷ 129.3			
% energy	33.9 ± 2.6	33.5; 32.1 ÷ 35.8	30–35	100.0	0.00
g/kg _bm_/day	1.46 ± 0.25	1.4; 1.3 ÷ 1.6	0.5–1.0		
Cholesterol (mg)	544.7 ± 245.9	447.9; 370.3 ÷ 715.3	<300	181.5	0.01
SFA (g)	31.15 ± 10.9	30.1; 25.9 ÷ 34.9			0.00
SFA (%)	9.7		<10 [ACSM]		0.58
PUFA (g)	18.9 ± 6.5	18.3; 13.3 ÷ 22.9	0.5–1.0 ^§^		0.00
PUFA (%)	5.9		6–10		0.87/0.00
MUFA (g)	43.5 ± 12.4	41.1; 34.6 ÷ 51.9			
MUFA (%)	13.6				
Omega-3FA (g)	3.4 ± 1,6	2.8; 2.0 ÷ 5.3			
Omega-3FA (%)	1.1		1–2		0.66/0.00
Omega-6 FA (g)	13.2 ± 4.9	11.5; 10.3 ÷ 16.2			
Omega-6FA (%)	4.1		5–8		0.02/0.00

Values are expressed as means ± SD and Me; Q1 ÷ Q3. CHO, carbohydrates; PRO, proteins; FAT, lipids; SFA, saturated fatty acids; PUFA, polyunsaturated fatty acids; MUFA, monounsaturated fatty acids. * European Food Safety Authority. ^§^ American Heart Association, American Dietetic of Canada.

**Table 3:** Dietary intake of vitamins and minerals in the windsurfers’ daily diet.

Vitamins and Minerals	Mean ± SD	Me; Q1 ÷ Q3	Recommendations ISSN	% RDA	*p*-Value
Sodium (mg/d)	2886.2 ± 724.7	2907.8; 2577.1 ÷ 3410.5	500 *	577.3	0.00
Potassium (mg/d)	4827.7 ± 1378.3	4273.7; 3749.4 ÷ 6344.9	2000 *	241.4	0.00
Calcium (mg/d)	991.5 ± 392.0	961.4; 621.0 ÷ 1056.0	1000 (ages 19–50)	99.2	0.95
Phosphorus (mg/d) (phosphate salts)	2068.8 ± 409.1	2050.9; 1794.8 ÷ 2381.4	700	295.5	0.00
Magnesium (mg/d)	471.9 ± 119.9	462.0; 352.0 ÷ 602.1	420 (Males)	112.3	0.19
Iron (mg/d)	16.0 ± 4.0	15.3; 11.9 ÷ 18.2	8 (ages 19–50)	200.0	0.00
Zinc (mg/d)	15.4 ± 3.5	14.6; 13.1 ÷ 17.6	11 (Males)	140.0	0.00
Vitamin A (mcg/d)	5204.1 ± 3099.0	3934.8; 2462 ÷ 7810.1	900 mcg/d (Males)	578.2	0.10
Vitamin D (mcg/d)	4.7 ± 3.0	4.1; 2.8 ÷ 5.1	5 (age < 51)	94.0	0.81
Vitamin E (mg/d)	15.5 ± 6.1	13.9; 10.3 ÷ 19.5	15	103.3	0.79
Vitamin C (mg/d)	202.5 ± 140	150.2; 109.2 ÷ 271.7	90 (Males)	225.0	0.02
Vitamin B1 (mg/d)	2.2 ± 1.2	1.7; 1.5 ÷ 2.4	1.2 (Males)	183.3	0.02
Vitamin B6 (mg/d)	3.3 ± 1.1	2.9; 2.3 ÷ 3.9	1.3 (age < 51)	253.8	0.00
Vitamin B12 (mcg/d)	5.6 ± 2.1	5.2; 4.5 ÷ 6.6	2.4	233.3	0.00

Values are expressed as means ± SD and Me; Q1 ÷ Q3. Recommended dietary allowances (RDA) based 2002 Food & Nutrition Board, National Academy of Sciences- National Research Council recommendations. * Estimated minimum requirement.

## Data Availability

The datasets generated during and/or analyzed during the current study are available from the corresponding author on reasonable request.
